# PReS-FINAL-2354: Laboratory investigation of the role of toll-like receptros on kidney cells in pathogenesis of lupus nephritis

**DOI:** 10.1186/1546-0096-11-S2-P344

**Published:** 2013-12-05

**Authors:** E Sen, G Welsh, A Ramanan, M Saleem

**Affiliations:** 1 Department of Paediatric Rheumatology, Bristol Royal Hospital for Children, UK; 2 Academic Renal Unit, University of Bristol, UK; 3 Department of Paediatric Nephrology, Bristol Royal Hospital for Children, Bristol, UK

## Introduction

Lupus nephritis (LN) is a serious complication of juvenile-onset systemic lupus erythematosus (JSLE). Current treatments include long-term immunosuppressants with significant side effects. There is a need to identify targets for more effective therapies. Toll-like receptors (TLRs) perform an important role in the innate immune response by recognising conserved molecules associated with pathogens. Previous studies have suggested a role for TLR7 and TLR9 in lymphocytes in the pathogenesis of SLE. Podocytes are specialised cells forming an important part of the glomerular filtration barrier. Biopsies from LN patients have demonstrated higher TLR7 and TLR9 expression in glomeruli compared with controls and suggest that these TLRs are localised to podocytes. We hypothesise that stimulation of TLR7 and/or TLR9 in podocytes, acting via nuclear factor kappa B (NFkB), leads to cellular damage and resultant kidney disease.

## Objectives

This research aims to examine the role of TLR7 and TLR9 in podocytes to identify potential targets for more effective therapies of lupus nephritis.

## Methods

Conditionally-immortalised human podocyte cell lines were cultured to examine expression of TLRs and the effects of their activation on the NFkB pathway and cell proliferation. Reverse transcription-quantitative polymerase chain reaction (RT-qPCR) and sodium dodecyl sulphate - polyacrylamide gel electrophoresis (SDS-PAGE) with Western blotting were used to detect TLR7 and TLR9 expression at the mRNA and protein level respectively. The effects of lipopolysaccharide (LPS), an inflammatory stimulus, on TLR expression were examined. Podocytes were stimulated with imiquimod, a specific TLR7 agonist and CpG oligodeoxynucleotide (ODN) 2216, a specific TLR9 agonist. Phosphorylation of NFkB pathway components was assessed with Western blotting and a cell proliferation assay used to estimate cell survival following treatments. The effects of treatment with TLR agonists in combination with dexamethasone were also examined.

## Results

Treatment of podocytes with LPS was associated with increased expression of TLR7 at both mRNA and protein levels whereas there was comparatively little change in TLR9. Exposure of the cells to imiquimod or CpG ODN 2216 increased phosphorylation of NFkB. Initial results from the cell proliferation assay suggested lower levels after imiquimod or CpG ODN 2216 treatment for 24 hours. Since podocytes are terminally differentiated this implies reduced cell numbers or lower metabolic activity. Exposure of the podocytes to dexamethasone was associated with lower expression of TLR7 and TLR9 at the mRNA level. There was preliminary evidence of less phosphorylation of NFkB when cells were treated with dexamethasone prior to TLR agonists compared with the latter alone.

## Conclusion

This study suggests that podocytes express TLR7 and TLR9. Agonists of these receptors have effects on intracellular signalling. If confirmed through ongoing work, the TLR-NFkB pathway in kidney cells may be a potential target for novel therapies in lupus nephritis.

## Disclosure of interest

E. Sen Grant/Research Support from: Arthritis Research UK, G. Welsh: None Declared, A. Ramanan: None Declared, M. Saleem: None Declared.

